# Combination treatment of prostate cancer with FGF receptor and AKT kinase inhibitors

**DOI:** 10.18632/oncotarget.14049

**Published:** 2016-12-20

**Authors:** Shu Feng, Longjiang Shao, Patricia Castro, Ilsa Coleman, Peter S Nelson, Paul D Smith, Barry R Davies, Michael Ittmann

**Affiliations:** ^1^ Department of Pathology and Immunology, Baylor College of Medicine and Michael E. DeBakey Department of Veterans Affairs Medical Center Baylor College of Medicine, Houston, 77030, TX, USA; ^2^ Division of Human Biology, Fred Hutchinson Cancer Research Center, Seattle, WA, 98109, USA; ^3^ Oncology iMED, AstraZeneca, Alderley Park, Macclesfield, UK

**Keywords:** prostate cancer, signal transduction, fibroblast growth factors, kinase inhibitor, AKT

## Abstract

Activation of the PI3K/AKT pathway occurs in the vast majority of advanced prostate cancers (PCas). Activation of fibroblast growth factor receptor (FGFR) signaling occurs in a wide variety of malignancies, including PCa. RNA-Seq of castration resistant PCa revealed expression of multiple FGFR signaling components compatible with FGFR signaling in all cases, with multiple FGF ligands expressed in 90% of cases. Immunohistochemistry confirmed FGFR signaling in the majority of xenografts and advanced PCas. AZD5363, an AKT kinase inhibitor and AZD4547, a FGFR kinase inhibitor are under active clinical development. We therefore sought to determine if these two drugs have additive effects in PCa models. The effect of both agents, singly and in combination was evaluated in a variety of PCa cell lines *in vitro* and *in vivo*. All cell lines tested responded to both drugs with decreased invasion, soft agar colony formation and growth *in vivo*, with additive effects seen with combination treatment. Activation of the FGFR, AKT, ERK and STAT3 pathways was examined in treated cells. AZD5363 inhibited AKT signaling and increased FGFR1 signaling, which partially compensated for decreased AKT kinase activity. While AZD4547 could effectively block the ERK pathway, combination treatment was needed to completely block STAT3 activation. Thus combination treatment with AKT and FGFR kinase inhibitors have additive effects on malignant phenotypes *in vitro* and *in vivo* by inhibiting multiple signaling pathways and mitigating the compensatory upregulation of FGFR signaling induced by AKT kinase inhibition. Our studies suggest that co-targeting these pathways may be efficacious in advanced PCa.

## INTRODUCTION

Prostate cancer (PCa) is the most common visceral malignancy and the second leading cause of cancer deaths in men in the United States. Activation of the AKT pathway has been strongly linked both by correlative studies and mouse models to PCa progression and is a promising target in PCa [1] . Indeed, there is vast literature on this topic dating back to the first discovery of the PTEN gene, which is commonly altered in PCa, and leads to activation of AKT signaling [2]. To cite a recent example, comprehensive studies by Taylor et al. [3] have demonstrated potentially activating alterations in the PI3K/AKT pathway in the vast majority of metastatic PCa samples. Activation of the AKT pathway also synergizes with TMPRSS2/ERG (T/E) fusion gene in prostate carcinogenesis based on human correlative studies and studies in genetically engineered mouse models [4–6]. Studies in mouse models have shown that simultaneous activation of AKT and the ERK pathway leads to aggressive PCa [7].

Fibroblast growth factors (FGFs) are a family of 18 different polypeptide ligands which bind to four distinct FGF receptors (FGFR1–4) which have variable affinities for different FGFs. FGFR signaling is involved in a variety of biological and pathological processes. There is extensive evidence implicating FGFR signaling aberrations in subsets of almost all common and many uncommon malignancies [8, 9]. A recent analysis of 4835 solid tumors sequenced seeking novel therapeutic targets revealed genomic alterations of FGFRs in 7.1% of cancers [10]. Given that multiple drugs targeting FGFRs (with variable levels of specificity) are in active clinical development [8, 11], this finding implies that FGFR pathway alterations are currently one of the top drug targets in advanced cancers.

There is extensive evidence from studies of human tumor samples and animal models that FGFs and FGF receptors are important in PCa initiation and progression [12, 13]. Our published studies have identified ERK as a key downstream mediator of FGFR signaling in PCa [14, 15]. Furthermore, in recent studies, using an FGFR specific inhibitor, we have shown that *in vivo* the vast majority of ERK signaling in PCa *in vivo* is downstream of FGFR signaling and blocking FGFR signaling is associated with marked inhibition of tumor growth [15] .

Given the frequent alterations in both AKT and FGFR signaling in PCa and the evidence of non-redundant activities of these two kinases, we examined whether simultaneous inhibition of these two kinases might have additive effects on PCa tumor progression. AZD4547 is an FGF receptor kinase inhibitor [16] that is currently in early phase clinical trials in several cancers. It inhibits FGFR1–4, with higher doses required to inhibit FGFR4 [16]. AZD5363 is an AKT kinase inhibitor that inhibits AKT1, AKT2 and AKT3 that is also in early phase clinical trials in several cancers including PCa [17]. We therefore examined potential additive effects of these two drugs *in vitro* and *in vivo* in PCa models and examined the mechanisms involved in the additive effects that we observed with these two agents.

## RESULTS

### Increased FGF receptor signaling in advanced prostate cancer

**Figure 1:**
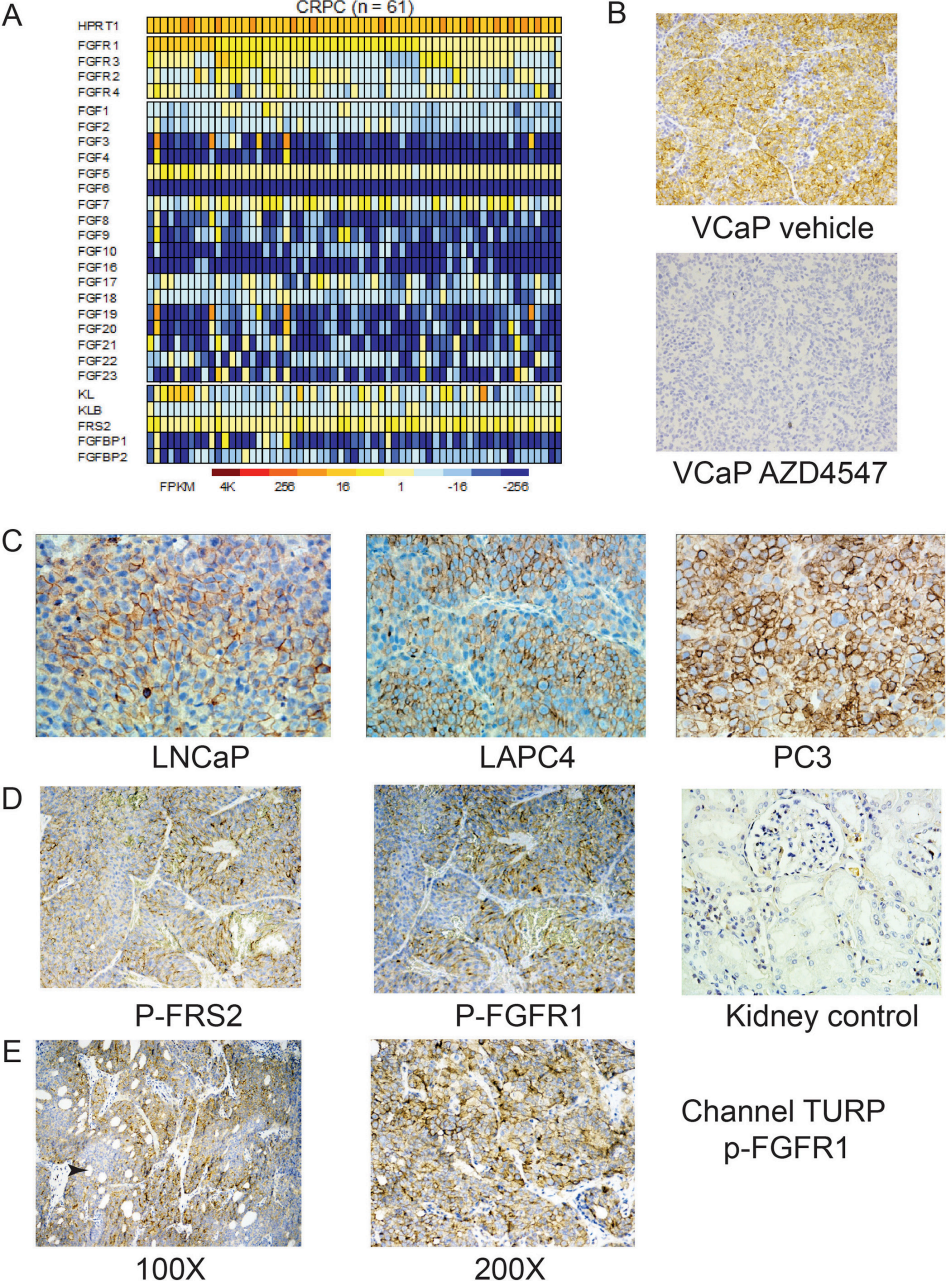
Increased FGFR signaling in advanced prostate cancer (**A**) Heat map of RNASeq analysis of components of the FGFR signaling system in 61 tumors from men with metastatic castration resistant prostate cancer is shown. Columns represent individual tumors and rows individual components of the FGFR signaling system. Expression in FPKM is indicated as shown in the scale. Transcripts with FPKM values of ≥ 1 were considered expressed. HPRT1 expression is shown for comparison and as a control. (**B**) Immunohistochemistry of VCaP xenografts with anti-phospho-FGFR1 (p-FGFR1) antibody showing membranous staining. Staining was abolished by pretreatment of mice with AZD4547. (**C**) Immunohistochemistry of prostate cancer cell line xenografts with p-FGFR1 antibody. Note strong membranous staining. (**D**) Immunohistochemistry of LuCaP xenograft with anti-phospho-FRS2 and anti-p-FGFR1 antibody. Kidney control from tissue microarray is shown, indicating that physiological FGFR signaling cannot be detected by this technique. (**E**) Transurethral resections from men with advanced prostate cancer showing membranous staining with anti-p-FGFR1 antibody. Heterogeneity of staining was noted, with a tendency for weaker staining in the center of tumor masses (arrow).

The FGFR signaling system is quite complex with 4 receptors and 18 ligands. Klotho proteins act as co-receptors for endocrine FGFs, which we have demonstrated to play a role in PCa [18, 19]. In addition, FRS2α acts as an obligate intracellular signal transduction molecule for transmitting signals from activated FGF receptors [20]. Finally, the FGF binding proteins can mobilize FGFs from extracellular stores and enhance FGF signaling. Thus multiple proteins can potentially increase FGFR signaling in PCa. To determine if the corresponding genes are expressed in castration resistant PCa we examined RNA-Seq data from 61 castration resistant PCa tumors. As shown in Figure 1A, all cancers expressed at least one FGFR and, in 27 cases, 3 or 4 receptors were expressed. All cases expressed FRS2α and 32 cases expressed KL or KLB endocrine FGF co-receptor. Sixty of 61 cases expressed one or more FGF ligands, with 55 of 61 cases expressing more than one ligand. Sixty cases expressed FGF5, 40 FGF7 and 38 expressed at least one other FGF ligand. Up to 10 FGF ligands were expressed in some cases. Finally, FGFBP1 and/or FGFBP2 were expressed in 7 of 61 cases. It should be noted that the multiple alterations observed in a single tumor can potentially have additive actions. Whether the FGF ligands are produced in an autocrine or paracrine manner (or both) is likely to be variable and will require further study.

To determine whether there is increased signaling from FGF receptors in PCa models established from advanced PCa, we evaluated FGFR signaling using two different antibodies for immunohistochemistry (IHC). The first antibody (p-FGFR1) recognizes a conserved site in FGFR1 that is phosphorylated in all 4 FGF receptors upon receptor activation, although it is not known whether this antibody has equal affinity for all four phosphorylated FGFRs when used in IHC. The second antibody (p-FRS2) recognizes phosphorylated FRS2α, which is the immediate downstream target of activated FGF receptors. As shown in Figure 1B, the anti-p-FGFR1 antibody stains VCaP xenograft tumors with a membranous pattern and staining is abolished in tumors from mice acutely treated with FGFR inhibitor AZD4547, confirming its specificity. Similar results were seen with the p-FRS2 antibody (Supplementary Figure 1A). IHC of xenografts from six PCa cell lines showed a similar pattern of staining (shown in Figure 1C and Supplementary Figure S1B). In addition, 27 of 41 LUCaP patient-derived xenografts showed variable activation of FRS2α signaling and 19 of 41 showed activation of FGFR1 when evaluated by IHC using tissue microarrays (Figure 1D). Of the 19 cases positive for p-FGFR1, 17 were also positive for p-FRS2α. Ten cases positive for p-FRS2α were not positive for p-FGFR1, indicating either activation of other upstream kinases or that the anti-p-FRS2α IHC is more sensitive than the anti-p-FGFR1 IHC. Finally, we examined six “channel TURPs” performed to relieve urinary tract obstruction in men with castrate resistant PCa using IHC for p-FGFR1 and p-FRS2α. These tissues were chosen since they are rapidly fixed in formalin (which should preserve the phosphorylated epitopes) and do not need to be decalcified (which may impact staining). Two of six cases showed strong but heterogeneous membranous staining with p-FGFR1 as shown Figure 1E. Two cases showed moderate membranous and cytoplasmic staining. IHC with p-FRS2 showed strong staining in 4 of 6 cases analyzed (Supplementary Figure S1C). Results are summarized in Supplementary Table S1. Our data supports the concept that significantly increased FGFR signaling occurs in advanced PCas.

### Additive effects of AKT and FGFR kinase inhibition *in vitro*

**Figure 2:**
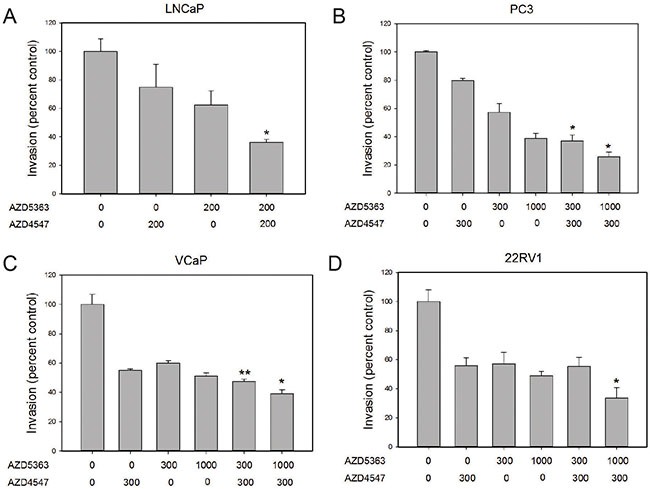
Additive effects of combination treatment on invasion Invasion through Matrigel was determined for the four cell lines shown in the presence of the indicated concentration (nM) of AZD4547, AZD5363 or both. Controls were treated with vehicle only. Data is expressed relative to vehicle only control (100%). Mean +/– standard error of the mean (SEM) is shown. All treatments except LNCaP with 200 nM AZD4547 showed statistically significant decreases from controls. Asterisks indicate statistically significant differences of combo treatment versus the same concentration of AZD5363; *p < .05; **< *p* <.01 by *t*-test.

All PCa cell lines tested had significant decreases in cell invasion when treated with either AZD4547 or AZD5363 (Figure 2). AZD4547 was used at 200 nM or 300 nM in order to inhibit FGFR4 (cellular IC_50_ 142 nM [16]). AZD5363 was used at doses that partially (300 nM) or almost totally (1000 nM) abolish AKT kinase activity in breast cancer and PCa cell lines [17]. In all cases additive effects were seen with combination treatments, with combination treatment resulting in 60–80% inhibition of invasion (Figure 2).

**Figure 3:**
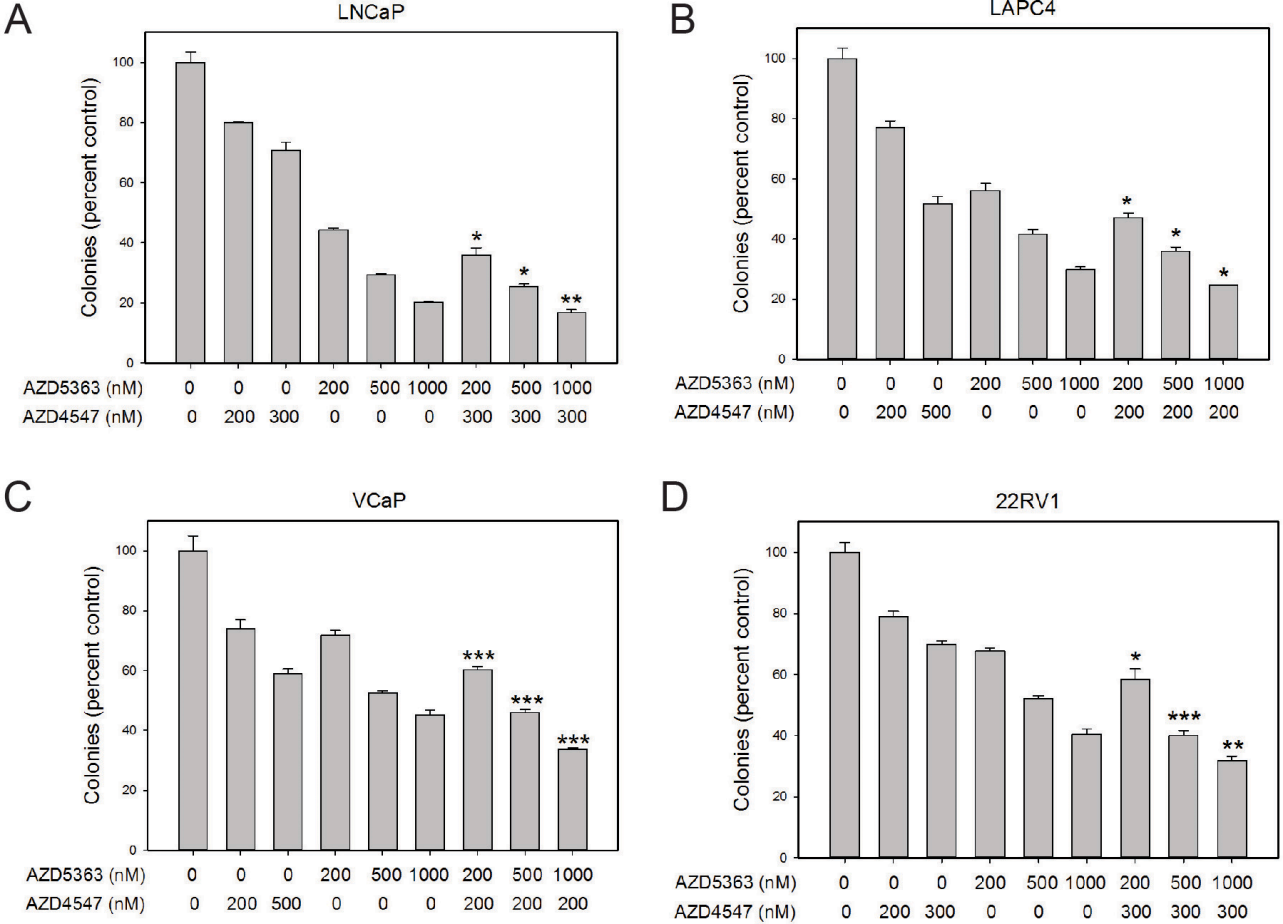
Additive effects of combination treatment on colony formation Colony formation in soft agar was determined for the four cell lines shown in the presence of the indicated concentration (in nM) of AZD4547, AZD5363 or both. Controls were treated with vehicle only. Data is expressed relative to vehicle only control (100%). Mean +/– standard error of the mean (SEM) is shown. All treatments showed statistically significant decreases from controls. Asterisks indicate statistically significant differences of combo treatment versus the same concentration of AZD5363; *p < .05; **< *p* <.01, ***p < .001 by *t*-test.

AZD5363 potently inhibited soft agar colony formation. AZD4547 also had significant effects on soft agar colony formation in all cell lines (Figure 3; see also Supplementary Figure S2). Additive effects of co-treatment with the two kinase inhibitors were seen in all cases.

### Additive effects of AKT and FGFR kinase inhibition *in vivo*

**Figure 4:**
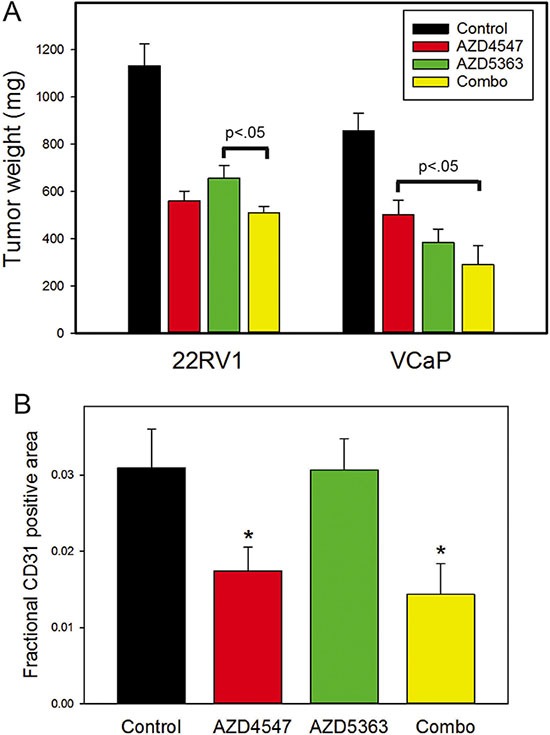
Combination treatment of prostate cancer xenografts (**A**) Xenografts of 22RV1 or VCaP prostate cancer cells were treated with AZd4547, AZD5363, both drugs or vehicle only. After 3 weeks (22RV1) or 4 weeks (VCaP) tumors were excised and weighed. Final tumor weights (mean +/– SEM) are shown. All treatment groups were statistically significantly decreased from controls by ANOVA (*p* < .001). Significant differences between treatment groups are indicated. (**B**) Angiogenesis was evaluated in tumors using anti-CD31 immunohistochemistry and image analysis. Fractional tumor area occupied by CD31 positive vessels is shown as mean +/– SEM. Statistically significant differences of treatment groups versus controls by *t*-test are shown; *p <.05.

To determine whether additive effects seen *in vitro* also occur *in vivo* we established subcutaneous xenografts in SCID mice using either 22RV1 or VCaP cells and treated the mice with AZD4547, AZD5363 or both drugs. Tumors were excised and weighed after 3 weeks (22RV1) or 4 weeks (VCaP). As shown in Figure 4A, combination treatment was more effective than either treatment individually for both cell lines. We had previously shown a significant decrease in angiogenesis in VCaP xenografts treated with FGFR inhibitor [15]. Examination of angiogenesis showed a significant decrease in angiogenesis in AZD4547 and combination treatment groups but not in the AZD5363 treated group (Figure 4B).

### Impact of combination treatment with AZD4547 and AZD5363 on cell signaling

**Figure 5:**
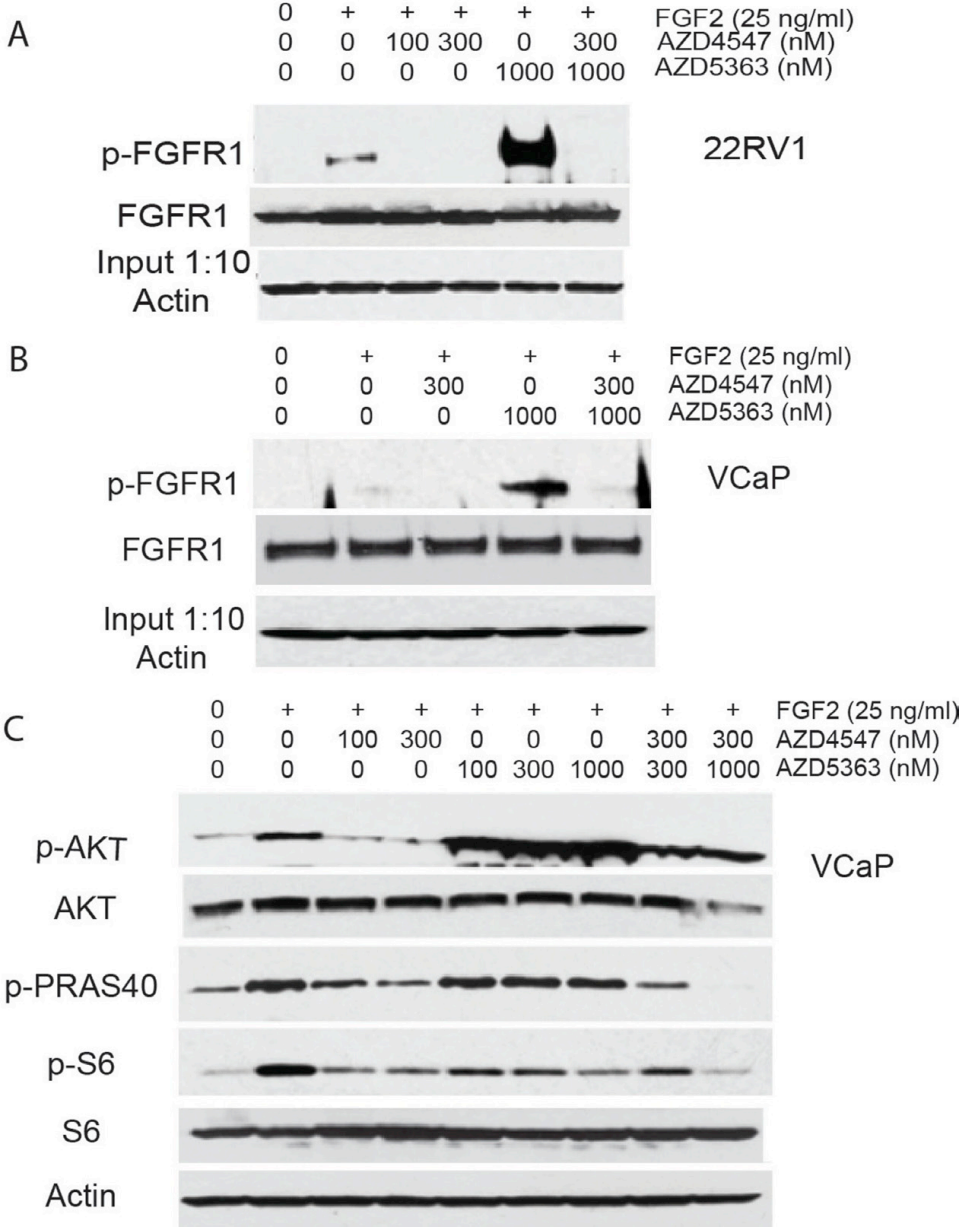
Upregulation of FGFR signaling by AZD5363 (**A** and **B**) 22RV1 (A) or VCaP (B) cells were pretreated with the indicated concentrations of AZD4547, AZD5363, both drugs or vehicle for 24 hrs in serum free medium. FGF2 was added and cell collected 15 minutes later. Protein lysates were analyzed by immunoprecipitation for FGFR1 phosphorylation and Western blot for total FGFR1. (**C**) Protein lysates from VCaP cell treated as in B, above, were analyzed by Western blot for phosphorylation of AKT (S473), pRAS40 (Thr246) and S6 ribosomal protein. Β-actin is loading control.

It has been shown that treatment with AZD5363 results in feedback upregulation of HER2 signaling in breast cancer [21] . We therefore sought to determine if AKT kinase inhibition can increase FGFR signaling in PCa cells. 22RV1 or VCaP cells were incubated for 24 hours in serum free medium in the presence of AZD4547, AZD5363, both drugs, or vehicle only. Cells were then treated with FGF2 and cell lysates prepared after 15 min treatment. Phosphorylation of FGFR1 was then assessed by immunoprecipitation and Western blot. As seen in Figure 5A and 5B, there was a marked increase in FGFR1 activation in cells treated with AZD5363, which was completely abolished by treatment with AZD4547. Of note, total FGFR1 was not increased under these conditions, implying alterations in ligand binding, FGFR1 transphosphorylation and/or stability of phospho-FGFR1. In similar experiments using VCaP cells, stimulation with FGF2 increased phosphorylation of AKT and its downstream targets PRAS40 and S6, all of which were abolished by treatment with AZD4547 (Figure 5C). Treatment with AZD5363 leads to hyperphosporylation of AKT due to feedback increases in AKT phosphorylation secondary to inhibition of its kinase activity, as has been described previously [22]. However, p-PRAS40 was minimally decreased and phosphorylation of S6 kinase was only partially blocked in these cells. It should be noted that AZD5363 is still effective in blocking AKT activity in these cells, since one would expect a marked in increase in both p-PRAS40 and p-S6 based on the marked increase in FGFR1 activity; the fact that it not increased or somewhat decreased indicates that the vast majority of AKT kinase activity was blocked, but sufficient activity is present under treatment to maintain partial activity. However, when cells were treated with both AZD5363 and AZD4547, phosphorylation of PRAS40 and S6 kinase was completely blocked at the higher dose of both drugs. Thus FGFR signaling can markedly increase AKT signaling that decreases the impact of AZD5363 on downstream AKT targets and this signaling can be inhibited by AZD4547.

**Figure 6:**
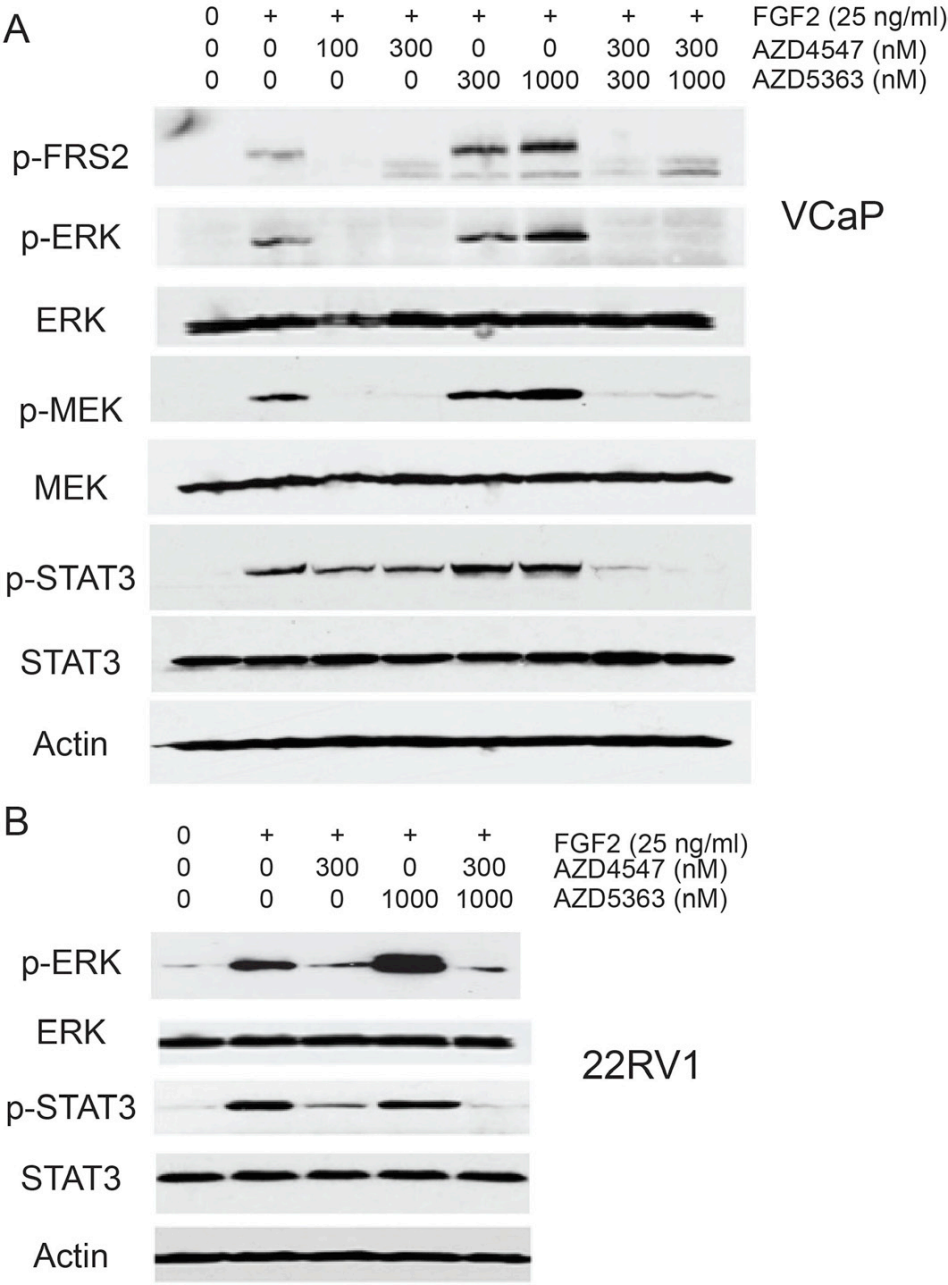
Impact of combination treatment on ERK and STAT3 signaling (**A**) VCaP (**B**) cells were pretreated with the indicated concentrations of AZD4547, AZD5363, both drugs or vehicle for 24 hrs in medium with 10% FBS. FGF2 was added and cells collected 15 minutes later. Protein lysates were analyzed by Western blot for phosphorylation of FRS2, ERK, MEK and STAT3. β-actin is loading control. (B) 22Rv1 cells were treated as for VCaP above and phosphorylation of ERK and STAT3 analyzed by Western blotting. β-actin is loading control.

We then carried out similar experiments using VCaP and 22RV1 cells in serum containing medium and evaluated other FGFR downstream targets. VCaP cells treated with AZD5363 and stimulated with FGF2 showed increased phosphorylation of FRS2 as well increased phosphorylation of both MEK and ERK relative to control cells stimulated with FGF2 (Figure 6A). ERK phosphorylation was also increased in 22RV1 cells treated with AZD5363 (Figure 6B). In addition, STAT3 phosphorylation was increased by FGF2 stimulation of VCaP and 22RV1 cells. Interestingly, this phosphorylation could only be partially blocked by AZD4547, implying that pretreatment with AZD4547 upregulated activity of bypass pathways that could activate STAT3 via other factors in serum. These pathways require AKT activity since incubation with both AZD4547 and AZD5363 completely blocked STAT3 phosphorylation. Consistent with this is the observation in both VCaP and 22RV1 cells that STAT3 phosphorylation was not increased to the same degree as ERK phosphorylation in cells preincubated with AZD5363, indicating that AKT kinase activity contributes to STAT3 phosphorylation independent of its effects on upregulating FGFR kinase activity.

## DISCUSSION

There is extensive literature indicating that FGFR signaling plays an important role in PCa [12, 13]. Our RNA-Seq analysis of the alterations in the complex FGFR signaling system in advanced PCa supports this concept. Our finding of extensive phosphorylation of FGFR1 and/or FRS2α in the majority of locally advanced, androgen independent PCa also supports this idea as does the finding of extensive FGFR phosphorylation in the majority of PDX lines (all established from advanced PCa). AZD4547 is a FGFR kinase inhibitor that has activity against an array of cancer types *in vitro* and *in vivo* [16]. Our prior studies have shown that AZ8010, a drug similar to AZD4547 but with inferior pharmacological properties, has *in vivo* activity against VCaP cells [15]. Our current studies show activity of AZD4547 against VCaP and 22RV1 cells in vivo. To date all PCa xenograft models tested, in a variety of microenvironments, including orthotopic, intratibial and subcutaneous, all show significant growth inhibition by AZD4547 *in vivo* (unpublished studies). This agrees with our IHC studies reported here that show extensive phosphorylation of FGFR1 and FRS2α in all xenograft tumors studied. Dovitinib, which targets FGFRs as well as several other receptor tyrosine kinases, showed significant activity in a clinical trial in advanced PCa [23]. It is currently unclear whether agents with more specific inhibitory activities such as AZD4547 will have similar efficacy in PCa compared to more non-specific agents, although it is likely they will have lower toxicity [11]. Thus AZD4547 appears to be a promising agent for treatment of advanced PCa.

Genomic changes activating AKT are among the most common alterations in human cancers, including PCa [3]. AZD5363 is an AKT kinase inhibitor that has been shown to have activity against many cancer types *in vitro* and *in vivo* [21, 22, 24]. Multiple publications have shown the efficacy of AZD5363 against a broad array of PCa cell lines *in vitro* and *in vivo* [22, 25–28]. In addition, AZD5363 has shown significant synergism with agents targeting androgen receptor signaling. We have confirmed significant *in vitro* and/or *in vivo* activity across a broad array of PCa cell lines (this manuscript and unpublished data). Currently early clinical trials of AZD5363 in men with advanced PCa and other cancer types are ongoing

**Figure 7:**
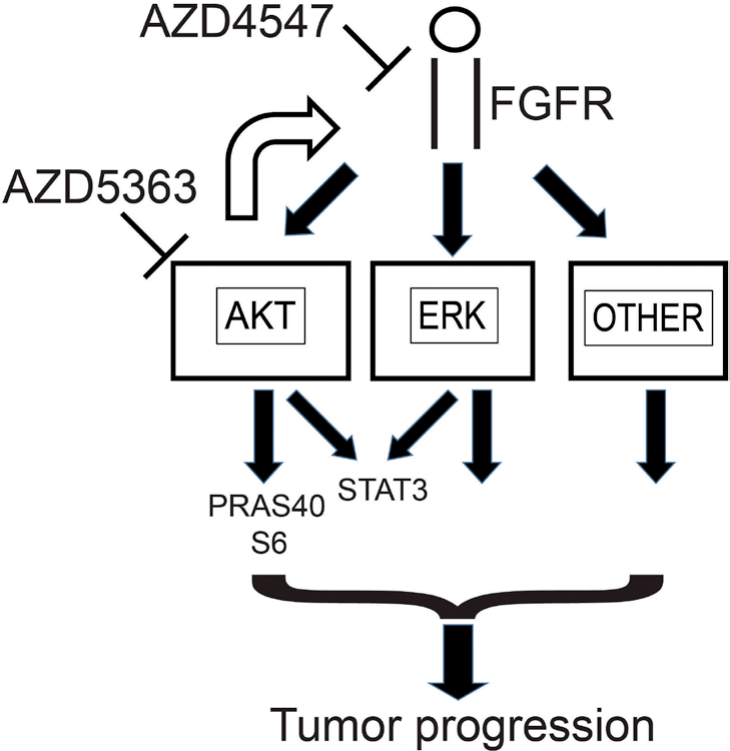
Summary of impact of AZD4547 and AZD5363 on cellular signaling in prostate cancer Ligand binding to FGF receptors activates multiple signaling pathways, including AKT, ERK and other pathways such as PLC-γ that can promote tumor growth and progression. AKT activation results in phosphorylation of downstream targets such as PRAS40 and S6. Phosphorylation of STAT3 at Ser727 is downstream of both ERK and AKT. When AKT is inhibited by agents such as AZD5363, this leads to feedback upregulation of FGFR signaling which tends to maintain phosphorylation of targets downstream of AKT and enhances activation of other pathways downstream of FGFR signaling. This can be prevented by inhibiting FGFR signaling with agents such as AZD4547.

Our studies demonstrate additive effects of AZD4547 and AZD5363 on PCa cell lines *in vitro* and *in vivo*. Three different mechanisms may account for these effects. First, FGFR signaling strongly enhances ERK activation. There is evidence from mouse models that AKT and ERK kinase activity can synergistically enhance PCa aggressiveness [7] and downstream targets of these two pathways have distinct biological activities. Other pathways are also activated by FGFR that are not downstream of AKT such as PLCγ and we have confirmed this in PCa (data not shown). Second, we observe strong upregulation of FGFR signaling when PCa cells are treated with AZD5363. This allows partial compensation for blocking AKT kinase activity by enhancing PI3K pathway activity upstream of AKT and/or enhancing the activity of ERK pathway as well as other pathways downstream of FGFR signaling. These findings are summarized in Figure 7. Finally, our data indicates that inhibition of FGFR signaling significantly inhibits angiogenesis, while inhibition of AKT has little impact on angiogenesis. It has been shown that AKT activity plays an important role in angiogenesis [29], but recent studies have shown that AKT3 (which is inhibited by AZD5363) can inhibit angiogenesis [30]. Thus inhibiting all three forms of AKT may have no net effect on angiogenesis. Further studies are needed to determine the extent to which inhibition of all three forms of AKT impacts angiogenesis in a variety tumor types and whether the net effect is context dependent. In contrast, AZD4547 inhibits angiogenesis in 22RV1 cells, in agreement with our prior results in VCaP cells [15]. FGFR signaling inhibition in endothelial cells should directly impact angiogenesis induced by FGFs. In addition, recent studies by Liu et al. [20] have shown that FRS2α signaling in PCa cancer cells indirectly enhances angiogenesis, at least in part by enhancing VEGF expression in the cancer cells. Thus co-treatment of PCa with both FGFR and AKT kinase inhibitors has additive effects due to direct effects, feedback upregulation and differing effects on the tumor microenvironment.

Our results are concordant with a number of recent studies in the literature. Davies et al. [24] reported that a bladder cancer cell line with activating mutations of both AKT and FGFR3 responded poorly to AZD5363 compared to breast cancer models with the same AKT mutation but responded significantly better to combination therapy with AZD4547 and AZD5363 compared to monotherapy with either agent. This result supports the concept that FGFR and AKT kinase activation can mediate cross resistance between these two pathways. Similarly, recent studies in lung and head neck cancer cell lines have known significant synergism when FGFR and MTOR signaling are simultaneously inhibited [31]. Finally, elegant studies in PTEN-deficient mouse models revealed significant decreases in tumor progression when treated with AZD5363 [28]. Such models are relevant since PTEN loss has been associated with better responses to AKT inhibition in human PCa [32] In this model IHC revealed focal areas of phosphorylated ERK and STAT3 in areas that maintained proliferative activity. This finding suggests focal activation of other pathways in these cancer cells that can maintain proliferation. While the nature of the activation signal(s) in this model is not known, the signaling pathways (ERK and STAT3) are the same as those observed in our studies of FGFR after treatment with AZD5363, indicating that *in vivo* it is possible for these pathways to maintain tumor proliferation.

Current clinical trials often seek to identify activation of the targeted pathway in patients prior to initiation of therapy in order to maximize chances of therapeutic benefit. A number of current trials of FGFR targeted agents use FGFR genomic alterations to determine if patients should be treated with FGFR targeted agents. Indeed, in cell lines and mouse models this approach has often been successful, with significant correlations between such genomic alterations and response to therapy [33]. However, this approach has some significant limitations, both in terms of sensitivity and specificity. Guagnano et al. [33] found that 47% of cell lines that responded to the FGFR kinase inhibitor NVP-BJG387 did not have genomic alterations in FGFRs. Some of these cell lines had increased FGF ligand expression. For example, liver cancers with both FGF19 amplification/overexpression and expression of β-klotho were sensitive to FGFR kinase inhibition. Overall, 37% of cancers sensitive to FGFR inhibition in this study did not have genomic lesions involving FGF ligands or FGFRs. Most of these cell lines showed increased FGFs and/or FGFR signaling without direct genomic lesions in these genes. Many cancers have increased expression of FGFRs that are associated with sensitivity to FGFR inhibitors but lack a corresponding genomic alteration, including lung cancer [31], head and neck cancer [31, 34], mesothelioma [35] and PCa [15]. FGFR gene amplification does not necessarily result in increased protein, and increased mRNA and protein is a better predictor of response to FGFR inhibitors than gene amplification [34, 36]. It has been shown that FGFR signaling can be an important downstream component of other genomic alterations in tumors, for example SNF5 deletion in malignant rhabdoid tumors [37]. Elegant studies in lung cancer based on large scale siRNA screens have shown that numerous alterations can increase FGFR signaling, most of which would not be predicted to do so [38]. As seen in our in silico analysis, numerous FGFR signaling components are expressed in PCa including receptors, co-receptors, FGF binding proteins and FGF ligands, with expression of multiple receptors and ligands in most cases. FRS2a, has been shown to be increased at the protein level by IHC in PCa [20] and such increased expression is correlated with aggressive disease. Similarly, FGFR1 and FGFR4 are both increased at the protein level in PCa and are associated with aggressive disease [39]. Multiple FGF ligands and co-receptors are also increased at the protein level in PCa as well [18, 19, 39–41]. Thus, while selection of patients with credentialed genomic alterations of FGFRs that are known to increase FGFR signaling is a rational approach, it fails to identify many patients who might potentially benefit from such therapy, including many with common cancers.

As an alternative to the genomic approach described above, IHC of phosphorylated FGFRs and/or FRS2α can potentially identify many different alterations in the complex FGFR signaling pathway that converge at FGFR phosphorylation and FRS2a activation. We have demonstrated increased phosphorylation of these phospho-epitopes in a variety of PCa PDX models and locally advanced, androgen independent PCa by IHC. While this approach is promising, there are issues that need to be resolved. For our FGFR phosphorylation studies we used an antibody raised against a phospho-epitope in FGFR1 that is highly conserved in FGFR2–4. It is likely that this antibody may recognize the analogous site in FGFR2–4 but this needs to be evaluated and the relative affinity for this site in all four FGFRs in IHC determined. It should also be noted that FRS2α phosphorylation is not specific for FGFR signaling. While several receptor tyrosine kinases can activate FRS2a, to date FGFR signaling appears to be the dominant pathway in PCa. In addition, the IHC is technically difficult and requires prolonged incubation with primary antibody. *In vitro*, FGFR and FRS2α phosphorylation is transient after ligand stimulation so it is unclear how sensitive these phospho-epitopes will be to preanalytical variables. However, Liu et al have shown increased phospho-FRS2α by IHC in clinically localized cancers [20]. Finally, it is possible that only those cases with extreme levels of FGFR signaling will be detected using this methodology and that tumors with lower levels of FGFR signaling may still be sensitive to FGFR inhibitors. Further studies are needed to resolve these issues.

In summary we provide evidence of additive biological effects of FGFR and AKT kinase inhibition in PCa and provide a molecular rationale for the additive effects seen. As such, co-treatment of these two targets is a rational approach in men with advanced PCa given that both of these pathways are commonly activated in advanced disease. It is likely that other cancers may also respond to co-targeting these two pathways. Ultimately clinical studies are needed to determine if patient outcomes are improved with this approach.

## MATERIALS AND METHODS

### Cell culture

Human PCa cells PC3, LNCaP and 22RV1 were maintained in RPMI-1640 medium (Invitrogen) supplemented with 10% fetal bovine serum (FBS, Invitrogen) and 100 ug/ml penicillin/streptomycin. VCAP and LAPC4 Cell lines were maintained in Dulbecco's Modified Eagle Medium (DMEM, Invitrogen) supplemented with 10% FBS and 1% penicillin/streptomycin (Invitrogen). PC346C cells were cultured in Dulbecco's modified Eagle's medium-Ham's F-12 medium ( Invitrogen) supplemented with 0.1 nm R1881, 2% fetal calf serum (PAN Biotech), 1% insulin-transferrin-selenium (Invitrogen), 0.01% BSA (Roche), 10 ng/ml epidermal growth factor (Sigma-Aldrich), 1% penicillin/streptomycin, 100 ng/ml fibronectin (Harbor Bio-Products), 20 μg/ml fetuin (ICN Biomedicals), 50 ng/ml cholera toxin (Sigma-Aldrich), 0.1 mm phosphoethanolamine (Sigma-Aldrich), and 0.6 ng/ml triiodothyronine (Sigma-Aldrich. PC346C cells were obtained from Martin Tenniswood, University of Albany. All other cell lines were obtained from the American Type Culture Collection. Cell were obtained between 2001 and 2012, expanded, frozen and stored as stocks in liquid nitrogen. Cells were discarded after 10 passages after revival. Mycoplasma testing was carried out monthly. All cell lines are authenticated by STR analysis at MD Anderson Cancer Center Characterized Cell Line Core Facility.

### Cell proliferation assays

AZD4547 and AZD5363 were provided by AstraZeneca. PCa cells were plated in to 96-well plates at 3×10^3^ cells per well in growth medium for 24 h before the drug treatment. Cells were then incubated with growth medium containing different concentrations of AZD4547, AZD5363, the combination of two drugs, or DMSO vehicle control for 72 h (LNCaP, LAPC4 and 22RV1) or 120h (VCaP). Cell proliferation was determined using the CellTiter 96 Aqueous One Solution Cell Proliferation Assay (Promega) as described by the manufacturer. The absorbance was read at 490 nm with VERSAmax Tunable microplate reader (Conquer Scientific).

### Matrigel invasion assays

Cell invasion assays were performed with BD BioCoat Matrigel invasion chambers (Becton Dickinson). After incubation with AZD4547, AZD5363, the combination of the two drugs, or vehicle only for 24 hours (PC3), 48 hours (LNCaP and 22RV1), or 72 h (VCaP), non-invading cells in the upper chambers were removed and the cells that penetrated through the matrigel to the lower surface of the filter were fixed and stained with Diff-Quik stain. The membranes were mounted on slides and scanned, photographed and all cells were counted. Each treatment was assayed in triplicate and three independent experiments were carried out.

### Soft agar colony formation assay

Cell suspensions of 3000 cells/ml were prepared in 0.35% agar were plated on a 0.6% agar foundation in 6-well culture plates at 37°C. After culture for 14 to 21 days, cells were stained with 2 mg/ml of p-iodonitrotetrazolium violet (Sigma) and colonies were counted with a dissecting microscope.

### Western blot

Total cellular protein lysate was prepared as described previously [19]. Briefly, cells were washed once with cold phosphate buffered saline and lysed in modified RIPA buffer containing Tris 50 mM, NaCl 150 mM, Triton X-100 1%, SDS 0.1%, deoxycholate 0.5%, sodium orthovanadate 2 mM, sodium pyrophosphate 1mM, NaF 50 mM, EDTA 5 mM, PMSF 1 mM and 1x protease inhibitor cocktail (Roche) and clarified by centrifugation. Protein concentration of the lysates was determined using BCA protein assay kit (Thermo Scientific). 40 μg of the extracted protein from each sample was subjected to electrophoresis in 7.5% or 10% sodium dodecyl sulfate (SDS) polyacrylamide gels. Proteins in the gels were transferred onto nitrocellulose membranes (Invitrogen) and subjected to Western blotting with different antibodies. Rabbit monoclonal antibodies from Cell Signaling Technology Inc were used including phospho-FRS2α (Tyr196); phospho-p44/42 MAPK (p-ERK1/2); p44/42 rabbit mAb; phospho-MEK1/2 (p-MEK1/2); MEK1/2; phospho-AKT (Ser473); total AKT; phospho-Stat3 (Ser727); phospho-PRAS40 (Thr246) and phospho-S6 ribosomal protein (Ser235/236) were all used at a 1:1000 dilution. The Stat3 mouse monoclonal antibody was from Cell Signaling and was also used at 1:1000 dilution. A goat polyclonal anti-b-actin antibody (Santa Cruz Biotech Inc.) was utilized at 1:5000 as loading control. After incubation with primary antibodies overnight at 4°C or for 1 hour at room temperature, horseradish peroxidase–labeled secondary antibodies were then applied to the membranes for 1 h at room temperature. Signals were visualized using enhanced chemiluminescence Western blotting detection reagents (Thermo).

### FGF receptor phosphorylation studies

To detect the effect of AZD4547, AZD5363 and the combination on phosphorylation of FGFR 1 in PCa cells, immunoprecipitation assays were performed. Briefly, protein extract (400 μg) of PCa cells treated with different drugs and/or FGF2 and DMSO vehicle control were precleared by incubating with 1 μg of normal rabbit IgG together with 20 μl of resuspended protein A/G Plus-Agarose (Santa Cruz Biotec, Inc.) at 4°C for 30 minutes and were subsequently incubated with 2 μg of anti-human FGFR-1 rabbit mAb (abCam EPR806Y) overnight at 4°C. 20 μl of resuspended protein A/G Plus-Agarose was then added to the lysate/antibody mixture. Following incubation for 1 hour at 4°C, the lysate/antibody/agarose mixture was centrifuged at 1000xg for 5 minutes at 4°C and the pellets were washed 4 times with 1.0 ml of RIPA buffer. Pellets were eluted in 40 μl of electrophoresis sample buffer and analyzed by Western blotting as described above with rabbit anti-phospho-FGFR mAb (1:1000, Cell Signaling). Parallel IP studies and Western blots were performed using anti-FGFR 1 mouse polyclonal antibody (Meridian Life Science Inc., P55213M) at 1:500 dilution.

### Xenograft studies

All experiments were carried out on 8–12 week-old male SCID mice. Each animal was injected subcutaneously with 1 × 10^6^ VCaP cells over both flanks. Two weeks later when the tumors were measureable (~3–5 mm^3^ in diameter?), those mice bearing subcutaneous tumors were divided into 3 experimental groups and 1 control group: experimental group I was treated with AZD4547 at 12.5 mg/kg/day in 1% polysorbate 80 by oral gavage; group 2 was treated with AZD5363 dissolved in vehicle (5% DMSO, 25% Kleptose HPB (Roquette), pH = 5) at 120 mg/kg/bid by oral gavage; group 3 ( the combined treatment group) was AZD4547 at 12.5 mg/kg/day + AZD5363 (120 mg/kg/bid). The control group was treated with vehicle only (1% polysorbate 80 and 25% Kleptose in water). Treatments were 5 days on and 2 days off. Four hours after the last treatment mice were euthanized and tumors were excised and weights and volumes measured. Portions of each tumor were fixed with buffered formalin, embedded in paraffin and processed for histological and IHC studies; the other portion was snap frozen in liquid nitrogen and protein were extracted for molecular studies. For 22RV1 cells, 5 × 10^5^ 22Rv1 cells were injected per site. The treatment was same as for the mice with VCaP xenograft. Mice were treated for a total of 3 weeks for 22RV1 xenografts and 4 weeks for VCaP xenografts. All procedures were approved by the Baylor College of Medicine Institutional Animal Use and Care Committee.

### Immunohistochemistry

The basic immunohistochemistry procedures were as described previously [14]. Antigen retrieval was carried out Tris-EDTA, ph 9.0 for 20 minutes in a rice steamer. Slides were stained with mouse monoclonal anti-phospho-FRS2α antibody (Cell Signaling Cat # 3861) at 1:30 dilution with overnight then for 1.5 hrs at 37° and 2.5 hrs at room temperature. For phospho-FGFR1 a rabbit polyclonal (Imgenex 6448A) was used at 1:100 dilution with procedures similar to the phospho-FRS2α except the 37° incubation was for 2 hours. To study angiogenesis 22RV1 xenografts a small tissue microarray was constructed. Angiogenesis was quantiated by image analysis after IHC with anti-CD31 antibody as described previously [15].

### RNA-Seq analysis

Samples were obtained from castration resistant PCa under an Institutional Review Board approved protocol and RNA prepared as described previously [42]. RNA concentration, purity, and integrity was assessed by NanoDrop (Thermo Fisher Scientific Inc) and Agilent Bioanalyzer. RNA-seq libraries were constructed from 1 ug total RNA using the Illumina TruSeq Stranded mRNA LT Sample Prep Kit according to the manufacturer's protocol. Barcoded libraries were pooled and sequenced six per lane on the Illumina HiSeq 2500 generating 50 bp paired end reads. Sequencing reads were mapped to the hg19 human genome using TopHat v2.0.12 [43]. Gene level abundance was quantitated from the filtered human alignments in R using the Genomic Alignments Bioconductor package [44]. Differential expression was assessed using transcript abundances as inputs to the edgeR Bioconductor package in R [45] which first corrects for transcripts per million, then normalizes for exonic size.

### Statistical analysis

Numerical values were compared using *t*-test (two sided) or ANOVA. Differences were considered significant if *p* < .05.

## SUPPLEMENTARY MATERIALS FIGURES AND TABLES


