# Supplementary material for: Combination treatment of prostate cancer with FGF receptor and AKT kinase inhibitors
Source: Oncotarget. 2016 Dec 20;8(4):6179–92. doi: 10.18632/oncotarget.14049 (PMC5351622; doi:10.18632/oncotarget.14049)
Supplement: Supplementary file 1 [file oncotarget-08-6179-s001.pdf]

Combination treatment of prostate cancer with FGF receptor and AKT kinase inhibitors

Supplementary Materials

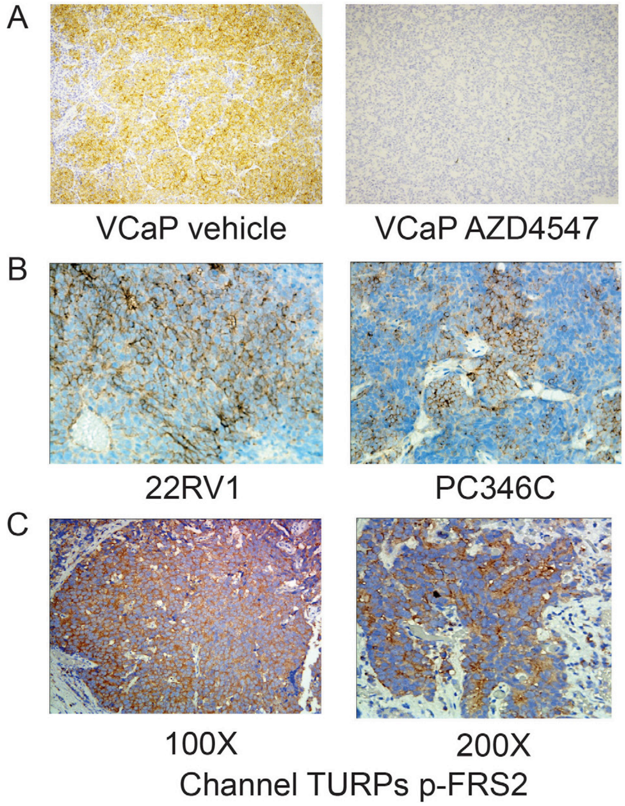

Supplementary Figure S1: (**A**) Immunohistochemistry of VCaP xenografts with anti-phospho-FRS2 antibody showing membranous staining. Staining was abolished by pretreatment of mice with AZD4547. (**B**) Immunohistochemistry of prostate cancer cell line xenografts with phospho-FGFR1 antibody. Note strong membranous staining. (**C**) Transurethral resections from men with advanced prostate cancer showing membranous staining with anti-phospho-FRS2 antibody.

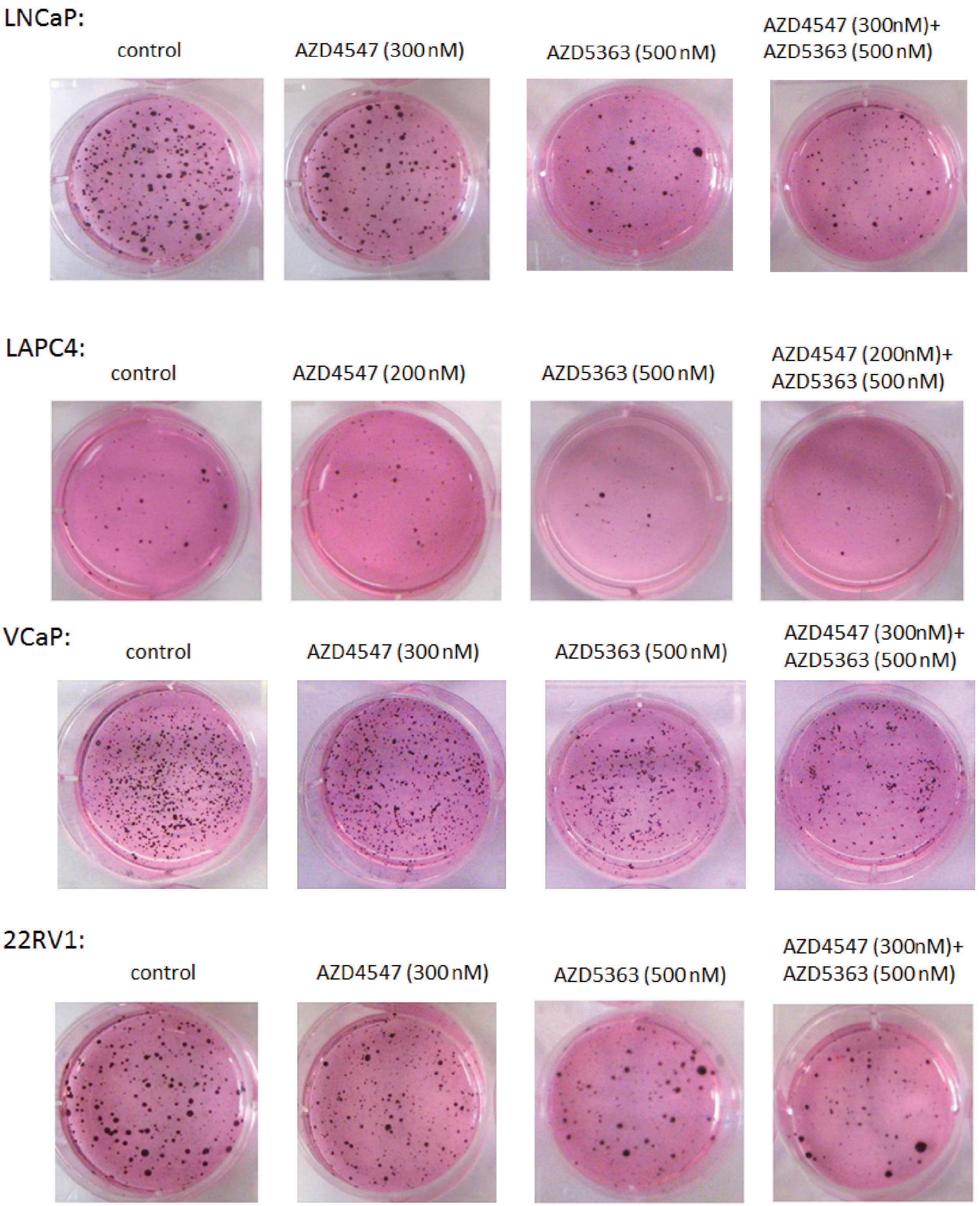

Supplementary Figure S2: Representative images of soft agar colonies for prostate cancer cell lines treated with indicated concentrations of AZD4547, AZD5363 or both drugs or vehicle only (control).

Supplementary Table S1: Summary of immunohistochemistry studies

	Anti-p-FGFR1 IHC	Anti-p-FRS2α IHC
**PCa cell line xenografts**	7 of 7	7 of 7
**LuCaP xenografts**	19 of 41	27 of 41
**Channel TURPS**	2 of 6	4 of 6
